# Diaqua­bis­(4-carb­oxy-2-propyl-1*H*-imidazole-5-carboxyl­ato-κ^2^ *N* ^3^,*O* ^4^)zinc(II) *N*,*N*-dimethyl­formamide disolvate

**DOI:** 10.1107/S1600536810022282

**Published:** 2010-06-16

**Authors:** Cheng-Jun Hao, Xiao-Jun Zhao

**Affiliations:** a College of Chemistry and Chemical Engineering, Pingdingshan University, Pingdingshan 467000, People’s Republic of China

## Abstract

In the crystal structure of the title compound, [Zn(C_8_H_9_N_2_O_4_)_2_(H_2_O)_2_]·2C_3_H_7_NO, the Zn^II^ atom is coordinated by two *N*,*O*-bidentate 2-propyl-1*H*-imidazole-4,5-dicarboxyl­ate anions and two water mol­ecules in a distorted octa­hedral environment. The asymmetric unit consists of one Zn^II^ atom located on a center of inversion as well as one anion, one water mol­ecule and one additional dimethyl­formamide mol­ecule that occupy general positions. Between the carboxyl and the carboxyl­ate group an intra­molecular hydrogen bond is found in which the hydroxy H atom is disordered. Disorder is also found for the H atoms of one of the three methyl groups. In the crystal structure, additional inter­molecular N—H⋯O and O—H⋯O hydrogen bonding is found.

## Related literature

For imidazole-4,5-dicarb­oxy­lic complexes, see: Maji *et al.* (2005 ▶); Yang & Zhang (2006 ▶).
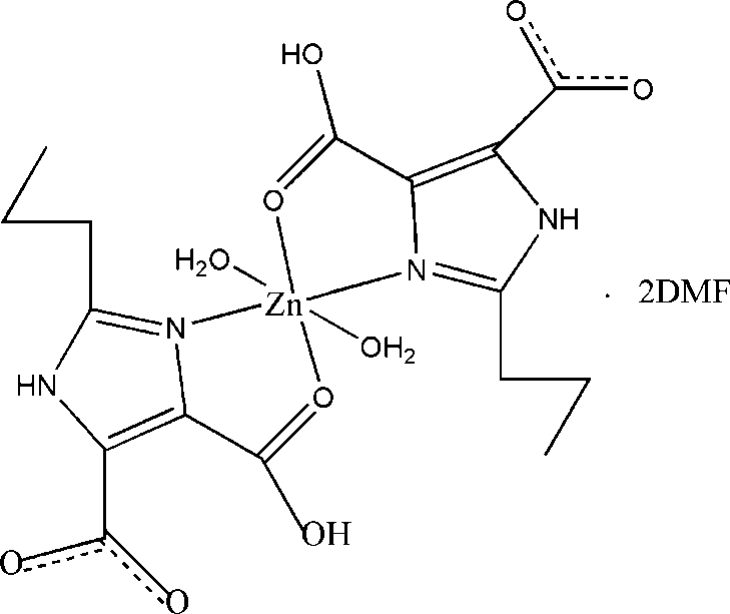


## Experimental

### 

#### Crystal data

[Zn(C_8_H_9_N_2_O_4_)_2_(H_2_O)_2_]·2C_3_H_7_NO
*M* *_r_* = 641.94
Triclinic, 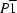

*a* = 7.3619 (9) Å
*b* = 9.3194 (13) Å
*c* = 11.2301 (15) Å
α = 76.281 (1)°
β = 87.621 (2)°
γ = 68.888 (1)°
*V* = 697.44 (16) Å^3^
*Z* = 1
Mo *K*α radiation
μ = 0.95 mm^−1^
*T* = 298 K
0.43 × 0.28 × 0.25 mm

#### Data collection

Bruker SMART 1000 CCD area-detector diffractometer
Absorption correction: multi-scan (*SADABS*; Bruker, 2007 ▶) *T*_min_ = 0.685, *T*_max_ = 0.797
3640 measured reflections
2425 independent reflections
2205 reflections with *I* > 2σ(*I*)
*R*_int_ = 0.015

#### Refinement

*R*[*F* ^2^ > 2σ(*F* ^2^)] = 0.029
*wR*(*F* ^2^) = 0.076
*S* = 1.06
2425 reflections
179 parameters
H-atom parameters constrained
Δρ_max_ = 0.26 e Å^−3^
Δρ_min_ = −0.33 e Å^−3^

### 

Data collection: *SMART* (Bruker, 2007 ▶); cell refinement: *SAINT* (Bruker, 2007 ▶); data reduction: *SAINT*; program(s) used to solve structure: *SHELXS97* (Sheldrick, 2008 ▶); program(s) used to refine structure: *SHELXL97* (Sheldrick, 2008 ▶); molecular graphics: *SHELXTL* (Sheldrick, 2008 ▶); software used to prepare material for publication: *SHELXTL*.

## Supplementary Material

Crystal structure: contains datablocks I, global. DOI: 10.1107/S1600536810022282/nc2185sup1.cif

Structure factors: contains datablocks I. DOI: 10.1107/S1600536810022282/nc2185Isup2.hkl

Additional supplementary materials:  crystallographic information; 3D view; checkCIF report

## Figures and Tables

**Table 1:** Hydrogen-bond geometry (Å, °)

*D*—H⋯*A*	*D*—H	H⋯*A*	*D*⋯*A*	*D*—H⋯*A*
O6—H2*O*6⋯O4^i^	0.82	2.16	2.9594 (14)	167
O6—H1*O*6⋯O4^ii^	0.82	1.98	2.796	175
O3—H1*O*3⋯O2	0.82	1.67	2.478	169
O2—H1*O*2⋯O3	0.82	1.66	2.478	177
N2—H1*N*1⋯O5^iii^	0.86	1.84	2.6789 (17)	166

Symmetry codes: (i) 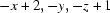; (ii) 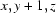; (iii) 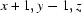.

**Fig. 1.**
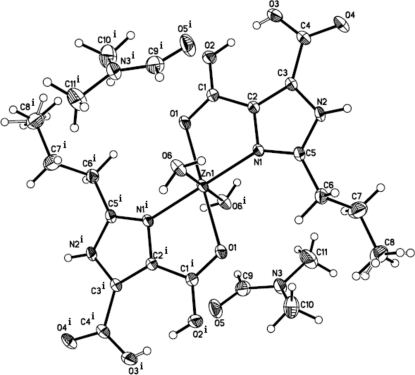
The structure of the title compound, showing the atomic numbering scheme. Non-H atoms are shown with 30% probability displacement ellipsoids (H atoms are represented by arbitrary spheres). Disordered H atoms are shown with full and open bonds. Symmetry codes: (i) 1-x, 1-y, 1-z.

**Fig. 2.**
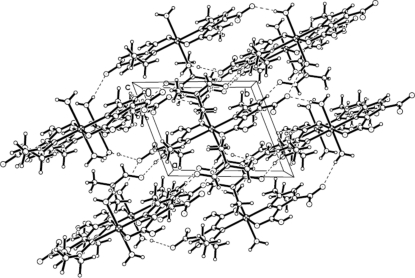
View of the three-dimensional network constructed by O—H···O and N—H···O hydrogen bonding interactions (the disordering is not shown for clarity and h<drogen bonding is shown as dashed lines)

## supplementary crystallographic information

### Comment

Imidazole-4,5-dicarboxylic acid (H_3_Imda) can be deprotonated to generate three types of anions, namely Imda^3-^, HImda^2-^ and H_2_Imda^-^and react with metal ions to form fascinating structures with different structures and useful properties (Maji *et al.*, 2005; Yang & Zhang, 2006). We have therefore reacted the 2-propyl-1*H* -imidazole-4,5-dicarboxylic acid with Zn(NO_3_)_2_ under hydrothermal conditions to obtain a new Zn^II^ complex and its structure is reported here. As illustrated in figure 1, the title complex molecule is a discrete complex, consisting of one Zn^II^ ion, two mono-deprotonated 2-Propyl-1*H*-imidazole-4,5-dicarboxy anions and two water molecules. The Zn ^II^ atom resides on a crystallographic inversion centre and is trans-coordinated by two N,*O*-bidentate 2-Propyl-1*H*-imidazole-4,5-dicarboxylate anions [Zn—O = 2.2052 (14) Å and Zn—N = 2.0643 (15) Å] and two water molecules [Zn—O = 2.1612 (15) Å], within a slightly distorted octahedral environment, with adjacent cis angles of [78.59 (6) °-101.41 (6) °]. The carboxyl and carboxylate group are via an intramolecular hydrogen bond in which the H atom is disordered. (Table 1). The crystal structure contain additional dimethylfromamide molecules that are linked to the complexes via N—H···O hydrogen bonding (Fig. 2). The complexes are additionally connected by intermolecular O—H···O hydrogen bonding between the carboxyl O atoms and the water H atoms (Table 1 and Fig. 2).

### Experimental

A mixture of Zn(NO_3_)_2_ (0.2 mmol, 0.03 g) and 2-propyl-1*H*-imidazole-4,5-dicarboxylic acid(0.5 mmol, 0.99 g) and 10 ml of C_3_H_7_NO was loaded in a 25 ml Telflon-lined stainless steel vessel and heated at 373k for 3 days. White crystals were obtained when the sample was cooled to room temperature slowly.

### Refinement

Carbon and nitrogen bound H atoms were placed at calculated positions and were treated as riding on the parent C or N atoms with C—H = 0.93 Å, N—H =0.86 Å, and with *U*_iso_(H) = 1.2 *U*_eq_(C, N). The O-H H atoms were located in difference map, their bond lengths set to ideal values and finally they were refined using a riding model with O—H = 0.85 Å. The H atom of the carboxyl group is disordered and was refined using a split model with sof of 0.6 and 0.4. The H atoms of one of the three methyl groups are also disordered and were refined using a split model with two orientations each rotated by 60° and sof of 0.6 and 0.4.

### Crystal data

**Table:**

[Zn(C_8_H_9_N_2_O_4_)_2_(H_2_O)_2_]·2C_3_H_7_NO	*Z* = 1
*M**_r_* = 641.94	*F*(000) = 336
Triclinic, *P*1	*D*_x_ = 1.528 Mg m^−^^3^
Hall symbol: -P 1	Mo *K*α radiation, λ = 0.71073 Å
*a* = 7.3619 (9) Å	Cell parameters from 2051 reflections
*b* = 9.3194 (13) Å	θ = 2.5–23.9°
*c* = 11.2301 (15) Å	µ = 0.95 mm^−^^1^
α = 76.281 (1)°	*T* = 298 K
β = 87.621 (2)°	Block, colorless
γ = 68.888 (1)°	0.43 × 0.28 × 0.25 mm
*V* = 697.44 (16) Å^3^	

### Data collection

**Table:**

Bruker SMART 1000 CCD area-detector diffractometer	2425 independent reflections
Radiation source: fine-focus sealed tube	2205 reflections with *I* > 2σ(*I*)
graphite	*R*_int_ = 0.015
φ and ω scans	θ_max_ = 25.0°, θ_min_ = 1.9°
Absorption correction: multi-scan (*SADABS*; Bruker, 2007)	*h* = −8→8
*T*_min_ = 0.685, *T*_max_ = 0.797	*k* = −10→11
3640 measured reflections	*l* = −13→10

### Refinement

**Table:**

Refinement on *F*^2^	Secondary atom site location: difference Fourier map
Least-squares matrix: full	Hydrogen site location: inferred from neighbouring sites
*R*[*F*^2^ > 2σ(*F*^2^)] = 0.029	H-atom parameters constrained
*wR*(*F*^2^) = 0.076	*w* = 1/[σ^2^(*F*_o_^2^) + (0.0389*P*)^2^ + 0.2536*P*] where *P* = (*F*_o_^2^ + 2*F*_c_^2^)/3
*S* = 1.06	(Δ/σ)_max_ < 0.001
2425 reflections	Δρ_max_ = 0.26 e Å^−^^3^
179 parameters	Δρ_min_ = −0.33 e Å^−^^3^
0 restraints	Extinction correction: *SHELXTL* (Sheldrick, 2008), Fc^*^=kFc[1+0.001xFc^2^λ^3^/sin(2θ)]^-1/4^
Primary atom site location: structure-invariant direct methods	Extinction coefficient: 0.067 (4)

### Special details

**Table:**

Geometry. All esds (except the esd in the dihedral angle between two l.s. planes) are estimated using the full covariance matrix. The cell esds are taken into account individually in the estimation of esds in distances, angles and torsion angles; correlations between esds in cell parameters are only used when they are defined by crystal symmetry. An approximate (isotropic) treatment of cell esds is used for estimating esds involving l.s. planes.
Refinement. Refinement of F^2^ against ALL reflections. The weighted R-factor wR and goodness of fit S are based on F^2^, conventional R-factors R are based on F, with F set to zero for negative F^2^. The threshold expression of F^2^ > 2sigma(F^2^) is used only for calculating R-factors(gt) etc. and is not relevant to the choice of reflections for refinement. R-factors based on F^2^ are statistically about twice as large as those based on F, and R- factors based on ALL data will be even larger.

### Fractional atomic coordinates and isotropic or equivalent isotropic displacement parameters (Å^2^)

**Table:**

	*x*	*y*	*z*	*U*_iso_*/*U*_eq_	Occ. (<1)
Zn1	0.5000	0.5000	0.5000	0.02921 (14)	
N1	0.6351 (2)	0.25671 (17)	0.54820 (14)	0.0248 (3)	
N2	0.8023 (2)	0.00172 (18)	0.60587 (15)	0.0287 (4)	
H1N1	0.8740	−0.0885	0.6506	0.034*	
N3	0.1215 (3)	0.4911 (2)	0.86419 (18)	0.0419 (4)	
O1	0.4380 (2)	0.43510 (15)	0.33379 (12)	0.0342 (3)	
O2	0.49486 (10)	0.22669 (8)	0.25454 (6)	0.0384 (4)	
H1O2	0.5591	0.1315	0.2727	0.058*	0.60
O3	0.68632 (10)	−0.06116 (8)	0.31714 (6)	0.0423 (4)	
H1O3	0.6132	0.0311	0.2920	0.063*	0.40
O4	0.86310 (10)	−0.23869 (8)	0.47855 (6)	0.0412 (4)	
O5	0.03879 (10)	0.74901 (8)	0.76828 (6)	0.0575 (5)	
O6	0.77394 (10)	0.51222 (8)	0.43114 (6)	0.0381 (4)	
H1O6	0.7938	0.5887	0.4435	0.057*	
H2O6	0.8631	0.4310	0.4647	0.057*	
C1	0.5133 (3)	0.2910 (2)	0.34026 (17)	0.0275 (4)	
C2	0.6268 (3)	0.1875 (2)	0.45418 (17)	0.0240 (4)	
C3	0.7305 (3)	0.0277 (2)	0.48902 (17)	0.0251 (4)	
C4	0.7647 (3)	−0.1017 (2)	0.42490 (19)	0.0306 (4)	
C5	0.7424 (3)	0.1403 (2)	0.63940 (17)	0.0272 (4)	
C6	0.7850 (3)	0.1559 (2)	0.76285 (18)	0.0357 (5)	
H6C	0.7435	0.2675	0.7620	0.043*	
H6D	0.9247	0.1093	0.7807	0.043*	
C7	0.6844 (4)	0.0768 (3)	0.8641 (2)	0.0553 (7)	
H7A	0.5456	0.1187	0.8432	0.066*	
H7B	0.7320	−0.0358	0.8679	0.066*	
C8	0.7156 (4)	0.0996 (3)	0.9888 (2)	0.0571 (7)	
H8B	0.7054	0.2071	0.9815	0.086*	0.60
H8C	0.8429	0.0289	1.0226	0.086*	0.60
H8A	0.6186	0.0775	1.0420	0.086*	0.60
H8E	0.8356	0.1177	0.9921	0.086*	0.40
H8D	0.7220	0.0065	1.0504	0.086*	0.40
H8F	0.6094	0.1893	1.0037	0.086*	0.40
C9	0.0112 (3)	0.6238 (3)	0.7883 (2)	0.0442 (6)	
H9	−0.0947	0.6218	0.7473	0.053*	
C10	0.2943 (4)	0.4853 (3)	0.9244 (3)	0.0616 (7)	
H10A	0.4070	0.4350	0.8825	0.092*	
H10B	0.3051	0.4261	1.0080	0.092*	
H10C	0.2858	0.5910	0.9226	0.092*	
C11	0.0897 (6)	0.3442 (3)	0.8741 (3)	0.0793 (10)	
H11A	−0.0320	0.3659	0.8325	0.119*	
H11B	0.0863	0.2947	0.9590	0.119*	
H11C	0.1939	0.2747	0.8372	0.119*	

### Atomic displacement parameters (Å^2^)

**Table:**

	*U*^11^	*U*^22^	*U*^33^	*U*^12^	*U*^13^	*U*^23^
Zn1	0.0353 (2)	0.01589 (19)	0.0328 (2)	−0.00545 (13)	−0.00206 (13)	−0.00454 (13)
N1	0.0288 (8)	0.0178 (8)	0.0263 (8)	−0.0071 (6)	−0.0014 (6)	−0.0037 (6)
N2	0.0310 (8)	0.0161 (8)	0.0325 (9)	−0.0048 (7)	−0.0042 (7)	0.0012 (7)
N3	0.0475 (11)	0.0272 (10)	0.0455 (11)	−0.0102 (8)	0.0006 (9)	−0.0037 (8)
O1	0.0437 (8)	0.0194 (7)	0.0312 (8)	−0.0033 (6)	−0.0080 (6)	−0.0020 (6)
O2	0.0494 (9)	0.0298 (8)	0.0317 (8)	−0.0069 (7)	−0.0092 (6)	−0.0090 (6)
O3	0.0549 (9)	0.0284 (8)	0.0421 (9)	−0.0081 (7)	−0.0025 (7)	−0.0156 (7)
O4	0.0415 (8)	0.0192 (7)	0.0586 (10)	−0.0040 (6)	−0.0032 (7)	−0.0113 (7)
O5	0.0633 (11)	0.0276 (9)	0.0651 (12)	−0.0046 (8)	−0.0201 (9)	0.0046 (8)
O6	0.0358 (8)	0.0230 (7)	0.0548 (10)	−0.0097 (6)	0.0027 (7)	−0.0097 (7)
C1	0.0283 (10)	0.0239 (10)	0.0281 (10)	−0.0075 (8)	0.0003 (8)	−0.0050 (8)
C2	0.0248 (9)	0.0205 (9)	0.0264 (10)	−0.0081 (7)	0.0016 (7)	−0.0051 (7)
C3	0.0245 (9)	0.0199 (9)	0.0300 (10)	−0.0077 (7)	0.0017 (7)	−0.0045 (8)
C4	0.0285 (10)	0.0229 (10)	0.0410 (12)	−0.0089 (8)	0.0050 (9)	−0.0100 (9)
C5	0.0291 (10)	0.0199 (9)	0.0296 (10)	−0.0079 (8)	−0.0023 (8)	−0.0011 (7)
C6	0.0445 (12)	0.0277 (11)	0.0320 (11)	−0.0117 (9)	−0.0089 (9)	−0.0020 (8)
C7	0.0699 (17)	0.0634 (17)	0.0420 (14)	−0.0321 (14)	0.0123 (12)	−0.0188 (12)
C8	0.0653 (17)	0.0591 (17)	0.0402 (14)	−0.0146 (14)	0.0039 (12)	−0.0123 (12)
C9	0.0379 (12)	0.0437 (14)	0.0437 (13)	−0.0057 (10)	−0.0053 (10)	−0.0099 (11)
C10	0.0469 (14)	0.0528 (16)	0.0639 (18)	−0.0051 (12)	−0.0138 (13)	0.0076 (13)
C11	0.113 (3)	0.0437 (17)	0.089 (2)	−0.0384 (18)	0.014 (2)	−0.0156 (16)

### Geometric parameters (Å, °)

**Table:**

Zn1—N1	2.0643 (15)	C1—C2	1.479 (3)
Zn1—N1^i^	2.0643 (15)	C2—C3	1.372 (3)
Zn1—O6^i^	2.1616	C3—C4	1.488 (3)
Zn1—O6	2.1616	C5—C6	1.485 (3)
Zn1—O1	2.2053 (14)	C6—C7	1.520 (3)
Zn1—O1^i^	2.2053 (14)	C6—H6C	0.9700
N1—C5	1.331 (2)	C6—H6D	0.9700
N1—C2	1.375 (2)	C7—C8	1.505 (3)
N2—C5	1.346 (2)	C7—H7A	0.9700
N2—C3	1.369 (2)	C7—H7B	0.9700
N2—H1N1	0.8600	C8—H8B	0.9600
N3—C9	1.322 (3)	C8—H8C	0.9600
N3—C10	1.443 (3)	C8—H8A	0.9600
N3—C11	1.448 (3)	C8—H8E	0.9602
O1—C1	1.240 (2)	C8—H8D	0.9601
O2—C1	1.284 (2)	C8—H8F	0.9602
O2—H1O2	0.8200	C9—H9	0.9300
O3—C4	1.274 (2)	C10—H10A	0.9600
O3—H1O3	0.8200	C10—H10B	0.9600
O4—C4	1.234 (2)	C10—H10C	0.9600
O5—C9	1.223 (3)	C11—H11A	0.9600
O6—H1O6	0.8201	C11—H11B	0.9600
O6—H2O6	0.8200	C11—H11C	0.9600
			
N1—Zn1—N1^i^	180.0	O3—C4—C3	116.34 (16)
N1—Zn1—O6^i^	92.14 (5)	N1—C5—N2	110.03 (17)
N1^i^—Zn1—O6^i^	87.86 (5)	N1—C5—C6	126.12 (17)
N1—Zn1—O6	87.86 (5)	N2—C5—C6	123.80 (17)
N1^i^—Zn1—O6	92.14 (5)	C5—C6—C7	113.04 (18)
O6^i^—Zn1—O6	180.0	C5—C6—H6C	109.0
N1—Zn1—O1	78.58 (5)	C7—C6—H6C	109.0
N1^i^—Zn1—O1	101.43 (5)	C5—C6—H6D	109.0
O6^i^—Zn1—O1	88.98 (4)	C7—C6—H6D	109.0
O6—Zn1—O1	91.02 (4)	H6C—C6—H6D	107.8
N1—Zn1—O1^i^	101.43 (5)	C8—C7—C6	113.9 (2)
N1^i^—Zn1—O1^i^	78.57 (5)	C8—C7—H7A	108.8
O6^i^—Zn1—O1^i^	91.02 (4)	C6—C7—H7A	108.8
O6—Zn1—O1^i^	88.98 (4)	C8—C7—H7B	108.8
O1—Zn1—O1^i^	180.0	C6—C7—H7B	108.8
C5—N1—C2	106.21 (15)	H7A—C7—H7B	107.7
C5—N1—Zn1	141.02 (13)	C7—C8—H8B	109.5
C2—N1—Zn1	112.56 (12)	C7—C8—H8C	109.5
C5—N2—C3	108.91 (16)	C7—C8—H8A	109.5
C5—N2—H1N1	125.5	C7—C8—H8E	109.5
C3—N2—H1N1	125.5	C7—C8—H8D	109.5
C9—N3—C10	119.9 (2)	H8E—C8—H8D	109.5
C9—N3—C11	121.1 (2)	C7—C8—H8F	109.5
C10—N3—C11	118.4 (2)	H8E—C8—H8F	109.5
C1—O1—Zn1	112.88 (12)	H8D—C8—H8F	109.5
C1—O2—H1O2	110.3	O5—C9—N3	124.6 (2)
C4—O3—H1O3	117.8	O5—C9—H9	117.7
Zn1—O6—H1O6	112.3	N3—C9—H9	117.7
Zn1—O6—H2O6	109.2	N3—C10—H10A	109.5
H1O6—O6—H2O6	108.9	N3—C10—H10B	109.5
O1—C1—O2	123.54 (16)	H10A—C10—H10B	109.5
O1—C1—C2	118.08 (17)	N3—C10—H10C	109.5
O2—C1—C2	118.37 (16)	H10A—C10—H10C	109.5
C3—C2—N1	109.67 (16)	H10B—C10—H10C	109.5
C3—C2—C1	132.62 (17)	N3—C11—H11A	109.5
N1—C2—C1	117.71 (16)	N3—C11—H11B	109.5
N2—C3—C2	105.17 (16)	H11A—C11—H11B	109.5
N2—C3—C4	122.91 (17)	N3—C11—H11C	109.5
C2—C3—C4	131.90 (18)	H11A—C11—H11C	109.5
O4—C4—O3	124.73 (17)	H11B—C11—H11C	109.5
O4—C4—C3	118.93 (18)		
			
N1^i^—Zn1—N1—C5	23 (100)	O1—C1—C2—N1	2.6 (3)
O6^i^—Zn1—N1—C5	−94.0 (2)	O2—C1—C2—N1	−175.99 (15)
O6—Zn1—N1—C5	86.0 (2)	C5—N2—C3—C2	0.5 (2)
O1—Zn1—N1—C5	177.5 (2)	C5—N2—C3—C4	−178.27 (17)
O1^i^—Zn1—N1—C5	−2.5 (2)	N1—C2—C3—N2	−0.2 (2)
N1^i^—Zn1—N1—C2	−151 (100)	C1—C2—C3—N2	−179.58 (19)
O6^i^—Zn1—N1—C2	92.28 (12)	N1—C2—C3—C4	178.35 (18)
O6—Zn1—N1—C2	−87.72 (12)	C1—C2—C3—C4	−1.0 (4)
O1—Zn1—N1—C2	3.76 (12)	N2—C3—C4—O4	−0.5 (3)
O1^i^—Zn1—N1—C2	−176.24 (12)	C2—C3—C4—O4	−178.83 (17)
N1—Zn1—O1—C1	−2.53 (13)	N2—C3—C4—O3	178.75 (15)
N1^i^—Zn1—O1—C1	177.47 (13)	C2—C3—C4—O3	0.4 (3)
O6^i^—Zn1—O1—C1	−94.92 (13)	C2—N1—C5—N2	0.4 (2)
O6—Zn1—O1—C1	85.08 (13)	Zn1—N1—C5—N2	−173.60 (14)
O1^i^—Zn1—O1—C1	168 (100)	C2—N1—C5—C6	−177.05 (18)
Zn1—O1—C1—O2	179.33 (13)	Zn1—N1—C5—C6	8.9 (3)
Zn1—O1—C1—C2	0.8 (2)	C3—N2—C5—N1	−0.6 (2)
C5—N1—C2—C3	−0.1 (2)	C3—N2—C5—C6	176.97 (17)
Zn1—N1—C2—C3	175.82 (12)	N1—C5—C6—C7	110.5 (2)
C5—N1—C2—C1	179.37 (16)	N2—C5—C6—C7	−66.6 (3)
Zn1—N1—C2—C1	−4.7 (2)	C5—C6—C7—C8	−176.5 (2)
O1—C1—C2—C3	−178.04 (19)	C10—N3—C9—O5	−3.7 (4)
O2—C1—C2—C3	3.3 (3)	C11—N3—C9—O5	−174.1 (2)

Symmetry codes: (i) −*x*+1, −*y*+1, −*z*+1.

### Hydrogen-bond geometry (Å, °)

**Table:**

*D*—H···*A*	*D*—H	H···*A*	*D*···*A*	*D*—H···*A*
O6—H2O6···O4^ii^	0.82	2.16	2.9594 (14)	167
O6—H1O6···O4^iii^	0.82	1.98	2.796	175
O3—H1O3···O2	0.82	1.67	2.478	169
O2—H1O2···O3	0.82	1.66	2.478	177
N2—H1N1···O5^iv^	0.86	1.84	2.6789 (17)	166

Symmetry codes: (ii) −*x*+2, −*y*, −*z*+1; (iii) *x*, *y*+1, *z*; (iv) *x*+1, *y*−1, *z*.
